# Updated overall survival data and predictive biomarkers of autologous NK cells plus Sintilimab as second-line treatment for advanced non-small cell lung cancer

**DOI:** 10.3389/fimmu.2025.1595382

**Published:** 2025-05-21

**Authors:** Lin Jia, Naifei Chen, Xiao Chen, Chao Niu, Ziling Liu, Kewei Ma, Lei Yang, Yuguang Zhao, Wei Song, Jin Lu, Chen Chen, Xiaofeng Cong, Xu Wang, Yinghui Xu, Guozhen Cui, Zengguang Liu, Rongrong Chen, Huan Yin, Na Zhang, Jiuwei Cui

**Affiliations:** ^1^ Cancer center, The First Hospital of Jilin University, Changchun, China; ^2^ Department of Medical Center, Geneplus-Beijing Institute, Beijing, China; ^3^ Department of Medical Affairs, Genecast Biotechnology, Wuxi, China

**Keywords:** non-small cell lung cancer, second line therapy, autologous NK cells, sintilimab, immunotherapy

## Abstract

**Purpose:**

Combination strategies involving immune checkpoint inhibitors (ICIs) have been a prominent focus of research in the treatment of non-small cell lung cancer (NSCLC). Our prior findings demonstrated that the combination of autologous NK cells with the PD-1 antibody (Sintilimab), offered promising efficacy in NSCLC patients who failed the first-line platinum-based chemotherapy. Here, we present updated overall survival (OS) data from the final analysis, aiming to identify patient subgroups that derive maximal benefit from this therapeutic approach.

**Methods:**

Twenty NSCLC patients without driver gene mutations were enrolled and treated with a combination of autologous NK cells and Sintilimab every three weeks. Multicolor immunofluorescence staining was applied to evaluate static markers within the tumor microenvironment. Concurrently, dynamic assessments were conducted using next-generation sequencing and monitoring of PD-1/PD-L1 expression on NK cells to identify patient populations with favorable prognoses.

**Results:**

The median OS was 27.3 months (95% CI, 0.76 to 53.8), with six patients still alive at the follow-up cutoff. A significant correlation was observed between the CD56+PD-L1+ cellular phenotype and extended survival. Clearance of circulating tumor DNA (ctDNA) and an increased percentage of PD-L1+ NK cells following treatment was associated with significantly better survival outcomes. Notably, prolonged treatment exposure did not lead to increased toxicity.

**Conclusion:**

The combination of autologous NK cells with Sintilimab significantly enhances long-term survival in NSCLC patients without exacerbating adverse effects, presenting a promising strategy for future combination immunotherapy approaches in NSCLC treatment.

**Clinical trial registration:**

https://www.clinicaltrials.gov/ct2/show/NCT03958097, identifier NCT03958097.

## Introduction

Lung cancer remains the most prevalent and lethal malignancy in China, with recent statistics indicating approximately 1,060,600 new cases annually, accounting for 22.0% of all cancer diagnoses and resulting in roughly 733,300 deaths, or 28.5% of cancer-related fatalities (1). Non-small cell lung cancer (NSCLC) constitutes about 85% of all lung cancer cases (2). For patients with specific genetic mutations, targeted molecular therapies are standard, while immune checkpoint inhibitors (ICIs) have become the primary treatment option for individuals without driver gene mutations. ICIs can significantly improve survival rates in patients with high Programmed Death-Ligand 1 (PD-L1) expression, with five-year survival rates reaching up to 32% (3). However, due to limitations such as variable efficacy, immune-related adverse events, and primary and acquired resistance associated with immunotherapy, the exploration of combination immunotherapy strategies is continually underway (4).

In recent years, adoptive immunotherapy, particularly involving natural killer (NK) cells, has emerged as a promising approach (5). Recent clinical studies have shown that NK cells combined with ICIs can enhance the objective response rate (ORR) and extend progression-free survival (PFS) and overall survival (OS) in PD-L1+ advanced NSCLC patients compared to ICI monotherapy (6, 7). Our previous research demonstrated that autologous NK cells combined with Programmed Cell Death Protein 1 (PD-1) antibody (Sintilimab) resulted in a 45% ORR and a median PFS of 11.6 months in a second-line treatment setting for NSCLC patients without driver gene mutations (8). However, whether the PFS benefit observed with the combination of NK cells and Sintilimab will lead to an OS advantage, along with the long-term safety profile of this regimen, necessitates further investigation through extended follow-up.

In this article, we present updated long-term OS data and conduct analyses to identify the patient groups that would derive the greatest benefit from the treatment regimen. Through the application of both static and dynamic biomarkers, such as multiplex immunofluorescence to analyze tumor microenvironment (TME) characteristics, Next-Generation Sequencing (NGS), and evaluating of PD-1/PD-L1 expression on NK cells, we aim to identify patient populations with favorable prognoses.

## Methods

### Study design

Details of the study have been described previously (**Supplementary Figure S1**) (ClinicalTrial.gov Identifier: NCT03958097) (8). Briefly, 20 patients enrolled the trial, all the patients received Sintilimab 200mg and 3×10^9^ NK cells every 3 weeks until progressive disease, unacceptable toxicity, or withdrawal of consent. The study protocol was approved by institutional review board of first hospital of Jilin University study center, and the study was done in accordance with standards of Good Clinical Practice and the Declaration of Helsinki. All patients provided written informed consent before enrollment.

### Participants

Aged ≥18 years, with pathologically confirmed locally advanced or metastatic NSCLC who had failed the first line of platinum-based chemotherapy were eligible for the study. Patients with EGFR or ALK mutation were excluded. An Eastern Cooperative Oncology Group performance status of 0 or 1 and measurable disease according to Response Evaluation Criteria in Solid Tumors version 1.1. Patients previously received immunotherapy (ICIs, Chimeric Antigen Receptor T cells, antitumor vaccine etc.) were not included.

### Endpoints

The primary end point of the study was PFS. Secondary endpoints included ORR, OS, duration of response (DoR), disease control rate (DCR), clinical benefit rate (CBR) and safety. The exploratory predictive biomarkers were Tumor Mutational Burden (TMB), PD-1 and PD-L1 expression on NK cells.

### DNA extraction and library preparation

Cell-free DNA (cfDNA) was isolated using the QIAamp Circulating Nucleic Acid Kit (Qiagen, Hilden, Germany). The germline genomic DNA from white blood cells was isolated using the QIAamp DNA Blood Mini Kit (Qiagen, Valencia, CA, USA). The DNA concentration was measured using a Qubit fluorometer and the Qubit dsDNA HS (High Sensitivity) Assay Kit (Invitrogen, Carlsbad, CA, USA). The size distribution of the cfDNA was assessed using an Agilent 2100 BioAnalyzer and a DNA HS kit (Agilent Technologies, Santa Clara, CA, USA) as previously reported (9, 10).

Sequencing libraries were prepared using the KAPA Library Preparation Kit (Kapa Biosystems, Wilmington, MA, USA). A custom-designed panel covering ~1.5Mbp genome and targeting 1021 cancer-related genes was used for hybridization enrichment with DNA libraries, and then sequenced using Gene+ Seq 2000 instrument (GenePlus-Beijing) (9, 10).

### Targeted next-generation sequencing and genomic data analysis

After removing adapters and low-quality reads, the clean reads were mapped to the human reference genome (hg19) using BWA18 (version 0.7.12-r1039). Reads realignment and recalibration were performed with the Picard software MarkDuplicates (v4.0.4.0; Broad Institute, Cambridge, MA, USA). Somatic single nucleotide variants (SNVs) were determined by MuTect and NChot (Geneplus-Beijing, inhouse), small insertions and deletions (indels) were called using GATK (v3.6-0-g89b7209; Broad Institute), copy number alterations (CNVs) were detected with CONTRA (2.0.8), and structural variations (SVs) were identified with the algorithm NCsv (v0.2.3 Geneplus-Beijing, inhouse). All final candidate variants were manually verified with the integrative genomics viewer browser. Somatic alterations were filtered with matched patient’s blood cell control to remove germline mutations (9).

TMB was calculated as the number of somatic nonsynonymous SNVs and small insertions/deletions per Mb in the coding region (with Variant Allele Frequency (VAF) ≥0.03 for tumor tissue, and ≥0.005 for ctDNA, respectively). TMB-high patients were identified with ≥9 mutations/Mb using the top quartile threshold of 2,000 samples from the Geneplus database (9).

Molecular tumor burden index (mTBI) was calculated using the mean allele fraction of mutations in a mutation cluster with the highest cellular prevalence of circulating tumor DNA (ctDNA) at each time point sample. mTBI reflects the percentage of ctDNA detected in cfDNA and its changes can reflect the change of tumor burden at the molecular level (11).

### ctDNA molecular response evaluation

The ctDNA concentration was expressed in haploid genome equivalents (hGE) per mL of plasma (hGE/mL) and was calculated by multiplying the maximal VAF of detected mutations by the plasma cell-free DNA concentration and dividing by 3.3, with the assumption that each haploid genomic equivalent weighs 3.3 pg (12). The ΔctDNA was calculated as the ctDNA concentration at the on-treatment timepoint minus the baseline ctDNA concentration in our cohort. ctDNA clearance was defined as the absence of non-germline variants at the response timepoint.

### Multiplex immunofluorescence analysis

4–5 μm thickness sections were cut from tumor FFPE tissues. The specific steps are as follows: baking at 63°C for 1h, dewaxing at room temperature, antigen repair is completed in EDTA solution (pH=9.0) and cleaned by TBST buffer for three times. Then the markers (CD4, CD8, PD-1, PD-L1, CD56) was labeled and stained. Upon the completion of the staining, the slides were scanned using Vectra 3.0.5 (PerkinElmer, Massachusetts, USA) in accordance with the manufacturer’s instructions. Multispectral images were unmixed using spectral library built from images of single stained tissues using the inform Advanced Image Analysis software (inForm 2.3.0; PerkinElmer, Massachusetts, USA). 12–15 representative original multispectral images were selected to train the inForm software (tissue segmentation, cell segmentation, phenotyping tool, and positivity score). All the settings applied to the training images were saved within an algorithm for batch analysis of multiple original multispectral images of the same tissue. The positive percentage of CD4, CD8, PD-1, PD-L1 or CD56 were calculated by the number of CD4, CD8, PD-1, PD-L1 or CD56 positive cell divided by the total number of nucleated cells.

### Statistical analysis

In this exploratory analysis of OS, mOS and OS rates were estimated using the Kaplan-Meier method. The Hazard Ratios (HRs) and confidence intervals (CIs) were estimated using stratified Cox proportional hazard models. Patients without events were censored at the date they were last known to be alive. Analyses were performed on the data in the overall trial population. In the current extended follow-up (data cutoff, January 31, 2024), a sufficient number of events was reported to provide an estimate of mOS.

## Results

### Patient and treatment exposure

The study enrolled a total of 20 patients between May 2019 and October 2020. As of the analysis cutoff date (January 31, 2024), six patients remained alive, with four maintaining progression-free disease status. Among those who experienced disease progression, progression-free survival was 29.6 and 25.6 months, respectively. Of the patients who remained progression-free, one discontinued treatment owing to adverse events, while another withdrew for personal reasons. The remaining two patients continued to participate in the study treatment. The median follow-up duration for the trial, from the time of consent to the cutoff date, was 49.5 months, with a minimum follow-up of 39.9 months. Patients received a median of 12 cycles of NK cells treatment (range: 2–36 cycles) and 11.5 cycles of Sintilimab (range: 2–64 cycles). The discrepancy in the maximum number of cycles between NK cell infusions and Sintilimab administration was mainly because disruptions during the COVID-19 pandemic sometimes prevented timely NK cell infusions while Sintilimab treatment continued as planned.

### Efficacy assessments

The primary endpoint PFS, secondary endpoints included ORR, DoR, DCR, CBR have been reported previously (8). PFS was 11.7 months at the extended follow-up analysis, almost the same as previously reported (**Supplementary Figure S2**).

### Overall survival

**Figure 1:**
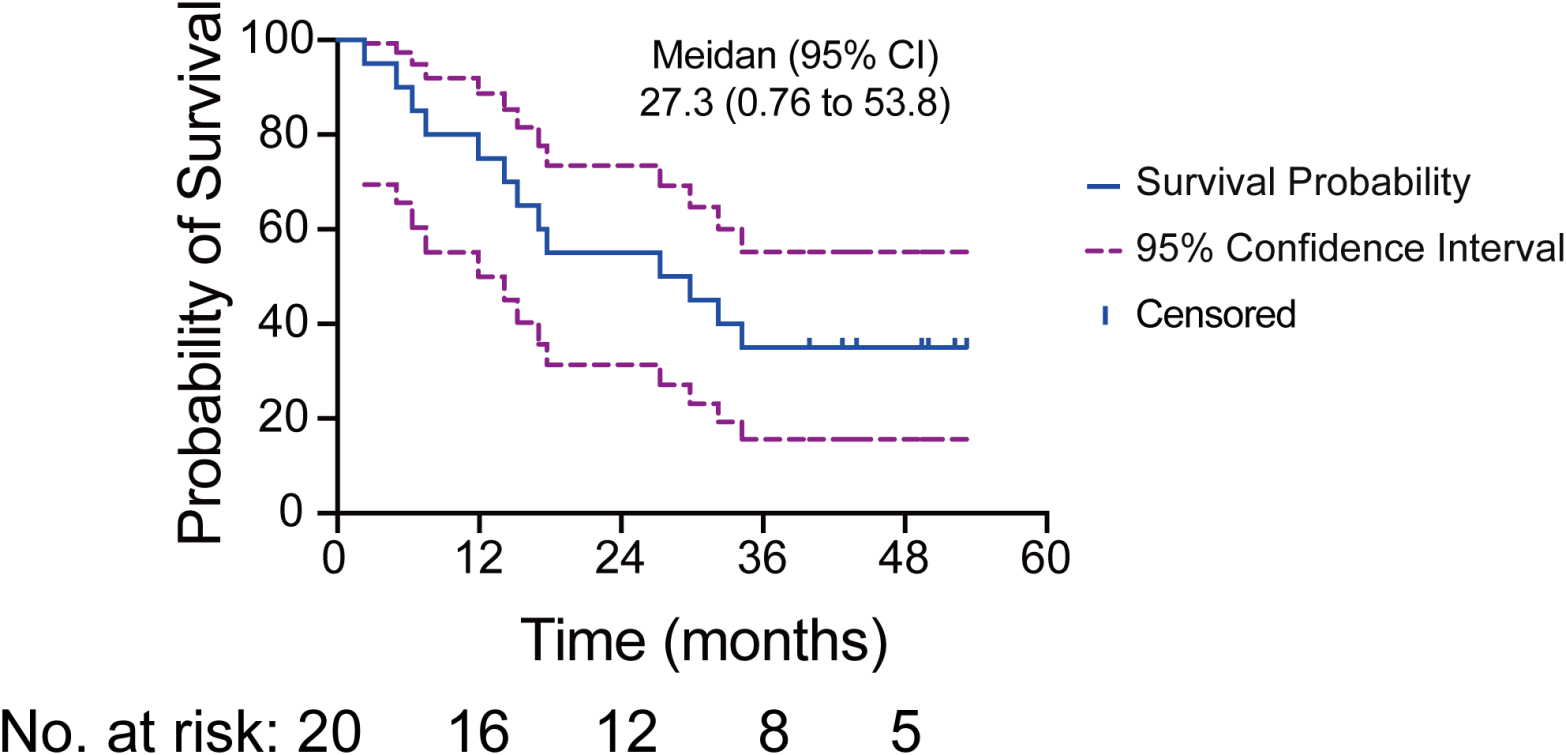
Kaplan-Meier curve illustrating the median OS and 95% CI for 20 patients. The solid blue line depicts the survival probability, while the dashed purple lines indicate the 95% CI. Tick marks represent censoring times. The numbers at risk correspond to the number of patients present at each time point.

The median OS was 27.3 months (95% CI, 0.76 to 53.8) in the intention-to-treat (ITT) population (**Figure 1**). OS was assessed across various subgroups, including age, smoking status, histology, and TMB level. No statistically significant differences were observed among these subgroups (**Supplementary Table S1**).

### Safety

With this extended follow-up analysis, no new safety events were observed (**Supplementary Table S2**). In conclusion, the adverse events (AEs) encountered by the ITT population corresponded with those delineated in the preceding report of this study. The most common grade 3 AEs observed were hyperglycemia, hypertriglyceridemia, creatine kinase increased, white blood cell decreased, and neutrophil count decreased.

### Dynamic predictive biomarkers

Liquid biopsy samples were collected at baseline and at each subsequent efficacy evaluation.

### ctDNA molecular response evaluation

**Figure 2:**
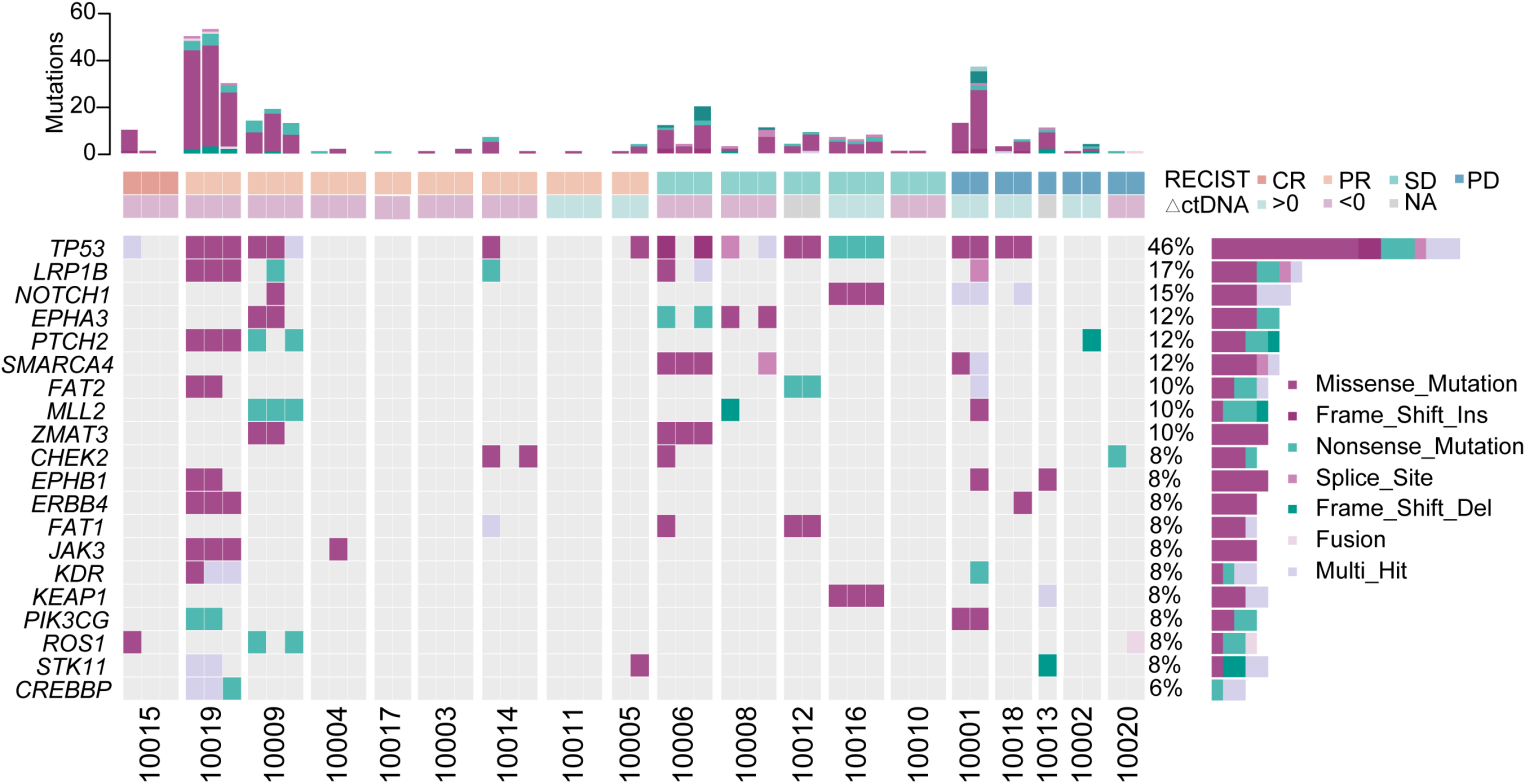
Overview of ctDNA gene mutations detected by NGS. The total number, distribution, and classifications of variants identified through liquid biopsy analyses in 19 patients are depicted. Sampling time points include baseline, after two treatment cycles, and the most recent clinical evaluation. Eighteen patients underwent at least two serial liquid biopsies, whereas patient 10013 only provided post-two-cycle sample. The top 20 most frequently mutated genes are listed, consistent with the established genomic landscape of NSCLC.

Among the 20 participants, liquid biopsy samples were obtained from 19 individuals. Of these, 18 patients had ctDNA measurements available for both baseline and post-baseline dynamics analyses. The top three identified mutated genes were TP53, LRP1 and NOTCH1.Notably, 7 out of 9 patients who achieved the best response of partial response (PR), or complete response (CR) exhibited decreased ctDNA levels during therapy. In contrast, 3 out of 4 patients who had the best response of progressive disease (PD) showed increased ctDNA levels at the first efficacy evaluation timepoint (**Figure 2**).

**Figure 3:**
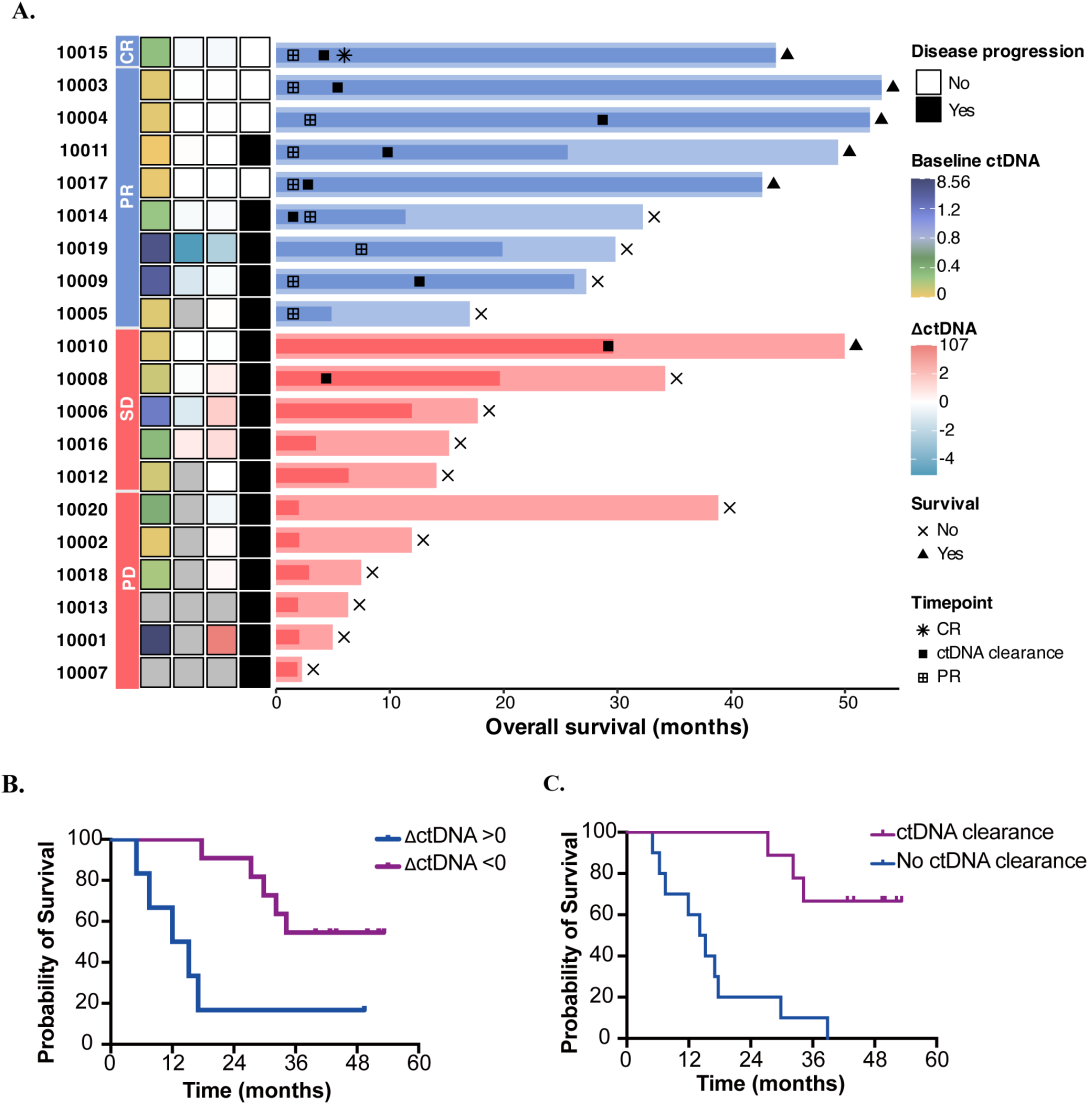
Analyses of PFS and OS by ctDNA dynamics. **(A)** Swimmer plot illustrating the timing of radiographic response assessment, PFS and OS for each evaluable patient. Patients are grouped by radiographic response category and ordered by OS within each group. The dark segment of each bar denotes PFS, while the bar’s total length represents OS. The three annotation columns to the left of the bars indicate ctDNA dynamics at baseline, at the response timepoint, and at the last sample. Gray tiles mark timepoints where no sample was available for analysis. The fourth annotation column represents disease outcome. **(B)** Patients exhibiting decreased ctDNA levels during therapy demonstrated longer OS (HR=0.23 [95%CI: 0.07-0.80]; P=0.021). **(C)** Patients achieved ctDNA clearance had longer OS compared those who never achieved ctDNA clearance (HR=0.11 [0.03-0.46]; P=0.002).

Through the analysis of PFS and OS in relation to ctDNA dynamics, we determined that changes in ctDNA levels (ΔctDNA) serve as a robust prognostic indicator. Median PFS for patients exhibiting decreased ctDNA levels was 26.2 months versus 3.2 months for patients with increased ctDNA (HR=0.19 [95%CI: 0.06-0.67]; P=0.01) (**Figure 3A**; **Supplementary Figure S3A**). Median OS for patients exhibiting decreased ctDNA levels was not achieved (54.5% of patients in this group were still alive) versus 13.6 months for patients with increased ctDNA (HR=0.23 [95%CI: 0.07-0.80]; P=0.021) (**Figures 3A, B**). Additionally, patients who achieved ctDNA clearance had significantly longer PFS (median PFS: 29.6 months vs 3.2 months; HR=0.08 [95%CI: 0.02-0.41]; P =0.002) and OS (median OS: NA vs 14.65 months; HR=0.11 [95%CI:0.03-0.46]; P=0.002) compared to those who never achieved ctDNA clearance (**Figure 3C**; **Supplementary Figure S3B**). Of particular note, patients who achieved ctDNA clearance demonstrated significantly prolonged survival outcomes, regardless of the timing of clearance or their best radiographic response, demonstrated significantly prolonged survival outcomes (**Figure 3A**). According to the latest ctDNA analysis, 3 out of 4 progression-free patients continue to show ctDNA clearance. The Response Evaluation Criteria in Solid Tumors (RECIST) were less effective in distinguishing patients with PR from those with stable disease (SD) in terms of PFS and OS, underscoring the heterogeneity of radiographic response assessments. In contrast, ctDNA more accurately reflects tumor burden dynamics. ΔctDNA demonstrated significant value in capturing survival outcomes. Furthermore, ctDNA analysis has revealed alterations in the mutational landscape in patients who develop resistance to combined NK cells and PD-1 monoclonal antibody (mAb) therapy (**Supplementary Figure S4B**).

### Dynamic mTBI evaluation

We previously reported that mTBI closely matches radiographic evaluations (8). Long-term monitoring of mTBI in patients has demonstrated a strong correlation between mTBI dynamics and radiographic changes. Specifically, a decline in mTBI levels is observed when patients achieve a radiographic response. Conversely, an increase in mTBI levels typically precedes tumor progression (**Supplementary Figure S4**).

### PD-1 and PD-L1 expression on NK cells

**Figure 4:**
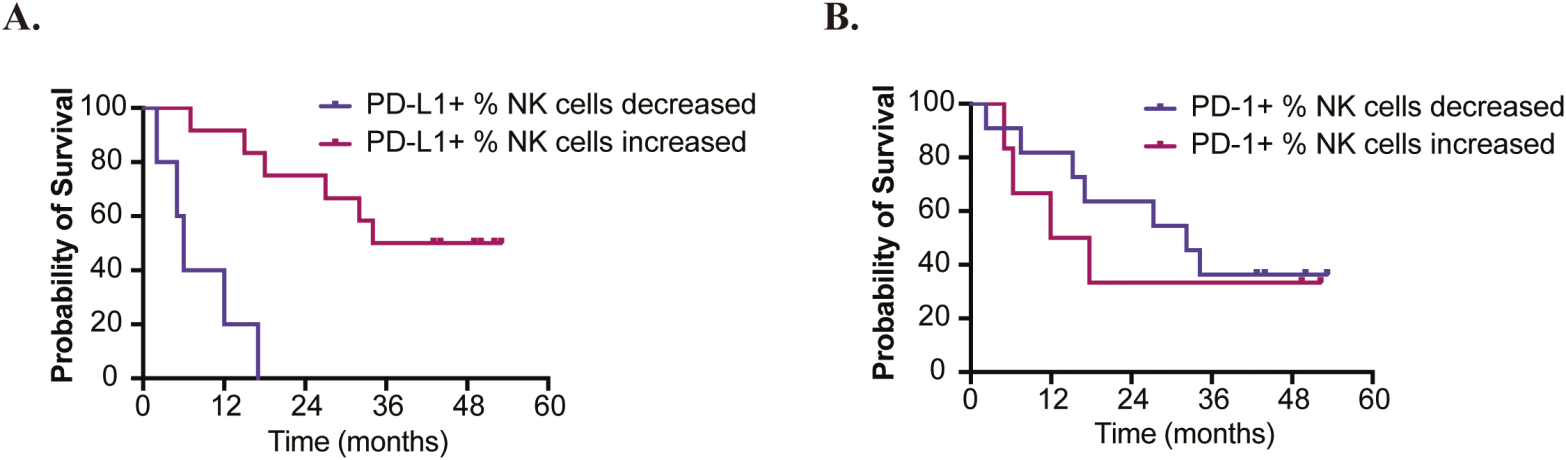
Analyses of OS based on changes in PD-1/PD-L1 Expression on NK Cells. **(A)** Patients who exhibited an increased percentage of PD-L1+ NK cells after treatment showed significantly longer overall survival (HR=0.07 [95%CI: 0.01-0.39]; P=0.002). **(B)** No significant difference in prognosis was observed between patients with increased versus decreased percentages of PD-1+ NK cells (HR=1.38 [95%CI: 0.40-4.73]; P=0.613).

We evaluated the expression of PD-1 and PD-L1 on NK cells. By comparing the long-survival group (>24months) to the short-survival group (≤24 months) (6), we found no significant differences in baseline PD-1 and PD-L1 expression on NK cells (**Supplementary Figures S5A, B**). However, patients who exhibited an increased percentage of PD-L1+ NK cells after treatment showed significantly longer OS compared to those with a decreased percentage (median OS: 43.5 months vs 6.0 months; HR=0.07 [95%CI: 0.01-0.39]; P=0.002). In contrast, no significant difference in prognosis was noted between patients with increased versus decreased percentages of PD-1+ NK cells (median OS: 14.8 months vs 32.2 months; HR=1.38 [95%CI: 0.40-4.73]; P=0.613) (**Figures 4A, B**).

### Static predictive biomarkers

Multiplex immunofluorescence analysis was successfully conducted on adequate tissue specimens obtained from eight patients: two with adenocarcinoma and six with squamous carcinoma. In terms of treatment outcomes, two patients exhibited best response of PD, three had SD, two achieved PR, and one attained CR. The median PFS for these patients was 15.8 months, and the median OS was 22.5 months.

**Figure 5:**
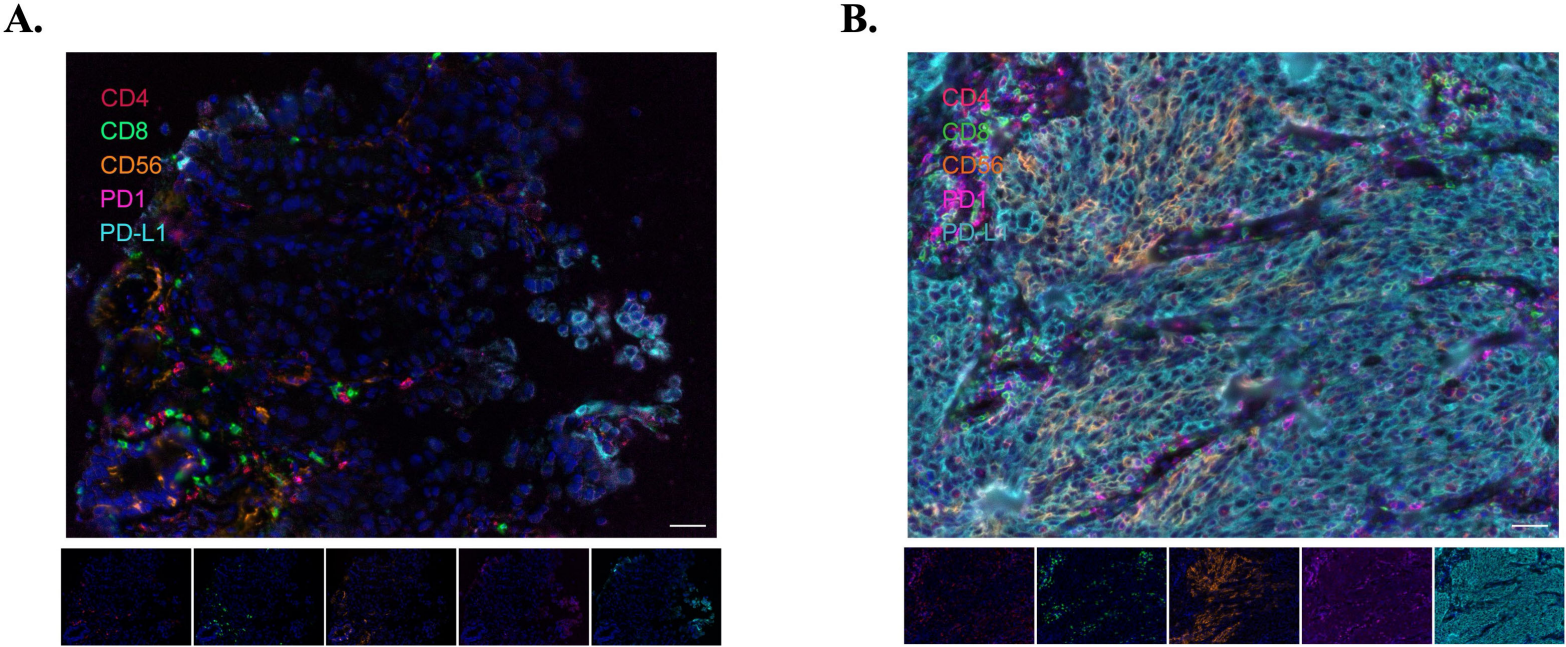
Representative immunofluorescence images illustrating immune cell infiltration in two patients. **(A)** Patient 10002, whose images reveal sparse immune cell infiltration, corresponding with a Best Overall Response (BoR) of PD. **(B)** Patient 10015, where the images demonstrate abundant immune cell infiltration, aligning with a BoR of CR. Scale bar represents 100µm.

**Figure 6:**
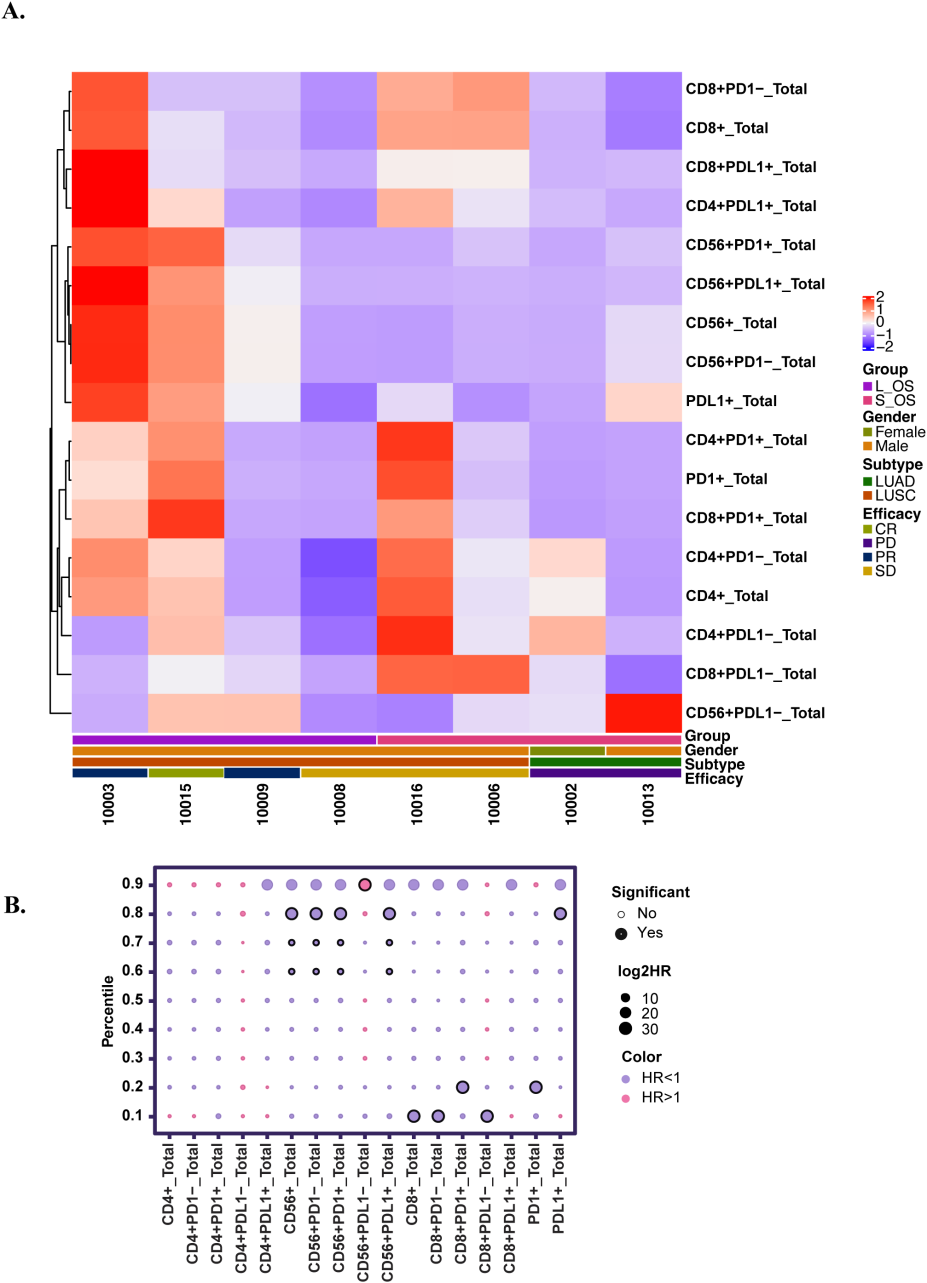
Comparative analysis of the relationship between cell type proportions and patients’ survival metrics. **(A)** Multifactorial heatmap. L_OS: long overall survival (>24months); S_OS: short overall survival (≤24 months); LUAD: Lung adenocarcinoma; LUSC: Lung squamous carcinoma **(B)** The bubble plot illustrates the comparison between different cell types and survival. The size of each bubble is proportional to the log2HR, indicating the magnitude of the effect. The color of the bubbles represents the direction of the effect: purple for HR<1, and pink for HR>1. Significant: P<0.05.

The analysis, which involved comparing the proportions of different cell types to patients’ survival, clearly facilitates the differentiation of patients based on their cellular phenotypes (**Figure 5**). To stratify patients into high and low groups for comparison, cutoff values are established at decile intervals. This approach yields bubble charts for various markers as depicted below, with HR and p-values organized into a table. Folders are created according to different deciles, and for each marker at each cutoff value, Kaplan-Meier survival curves were plotted and annotated with HR and p-values. The HR were calculated using the Cox proportional hazards model, while between-group comparisons were evaluated using the log-rank test (**Figure 6**). The analysis revealed that the presence of CD56+, CD8+, PD-1+, and PD-L1+ cells within the tumor microenvironment serves as a harbinger of a favorable therapeutic response, demonstrating a significant association with extended patient survival (**Supplementary Figure S6**). Notably, the CD56+PD-L1+ cellular phenotype emerged as a particularly potent predictor of beneficial treatment outcomes, consistently correlating with increased survival durations (**Supplementary Figures S6**, **S7**).

## Discussion

Our study demonstrates that combining NK cells with PD-1 mAb not only enhances short-term metrics, such as ORR and PFS, but also significantly extends the long-term metric of OS. In a cross-sectional comparison with the same term clinical studies, our research demonstrated longer OS data for second-line treatment of NSCLC without driver mutation genes. Hu et al. reported 7.5 vs. 3.3 months PFS for anti–PD-1 therapy combined with chemotherapy and/or bevacizumab (13). Wu et al. (CheckMate 078) noted an OS of 11.9 vs 9.5 months for nivolumab compared to docetaxel (14), and Rivalland et al. (RATIONALE-303) observed 17.2 vs 11.9 months OS for tislelizumab versus docetaxel (15). In contrast, our study showed a median PFS of 11.7 months and a median OS of 27.3 months when using NK cells combined with PD-1 inhibitors. Additionally, a Korean clinical study (SNK01 plus pembrolizumab) in previously treated NSCLC reported a 2-year survival rate of 58.3% compared to 16.7% in the control group (6), whereas the 2-year survival rate observed in our study was 55%. These cross-study comparisons suggest that, despite the lack of a direct control group, NK cells plus PD-1 mAb therapy may confer a survival benefit in NSCLC. Due to the limitations of ICIs immunotherapy—including variable efficacy, potential for immune-related adverse events, and challenges with primary and acquired resistance—there is an ongoing effort to explore various combination therapies involving ICIs. These combination strategies have become central in the quest to enhance the overall effectiveness of immunotherapy. However, existing studies have shown that improvements in PFS and OS are not particularly impressive, with some showing benefits in PFS without corresponding improvements in OS (16).

Furthermore, long-term administration of this treatment regimen does not increase adverse effects (**Supplementary Table S2**). The most frequently observed AEs included hypoalbuminemia, anemia, hypothyroidism, and hyperglycemia, with most AEs classified as grade 1–2 in severity. Our analysis revealed no additional AEs beyond those previously reported, and within the updated data from our study, there were no new or unexpected AEs identified, substantiating the favorable safety profile of the combined PD-1 mAb and NK cells therapy. Because patients’ performance status remained stable after the combined NK-cell and Sintilimab therapy, 8 of the 16 individuals (50%) who later exhibited disease progression were able to proceed to further anti-tumor treatment (**Supplementary Table S3**). Our study indicates that combining NK cells with PD-1 mAb provides long-term therapeutic benefits without additional toxicity, presenting a promising avenue for the future enhancement of combination immunotherapy strategies.

In addition, we investigated the correlation between indicators that reflect the depth of treatment response and OS. The RECIST v1.1 assessment, as a widely used standard for evaluating efficacy, can partially indicate the depth of response, patients who achieve a clinical response tend to have prolonged OS. Moreover, our analysis of dynamic ctDNA levels revealed a significant correlation between changes in ctDNA levels and patient prognosis. Specifically, a decrease in ctDNA levels was associated with longer OS compared to those with increased ctDNA levels. Notably, patients who achieved ctDNA clearance demonstrated significantly extended OS, as the clearance of ctDNA generally signifies a reduction in tumor burden. Among patients who achieved PR according to the RECIST v1.1 criteria, those who experienced ctDNA clearance had longer OS compared to those who did not achieve ctDNA clearance (median OS: NA vs 23.4 months; HR=0.09 [95%CI: 0.01-1.05]; P=0.055). A similar trend was observed in patients with SD, with OS of 42.1 months compared to 15.2 months, although this was not statistically significant (HR=0.01 [95%CI: 0.0-153.55]; P=0.360) due to the limited number of patients (**Supplementary Figure S8**). Through the analysis of these data, it is suggested that adding ΔctDNA evaluation to the existing RECIST criteria can better predict the prognosis of patients undergoing immunotherapy (17).

The quest to identify biomarkers that can predict the efficacy of immunotherapy has consistently been challenging, especially within the context of combination therapy, which currently faces substantial obstacles. In our study, we investigated predictive biomarkers for the efficacy of immunotherapy, including traditional PD-L1 expression and TMB levels (**Supplementary Table S1**). However, we found that their predictive effectiveness was limited. By conducting a more detailed analysis of the tumor microenvironment, we discovered that tumor-infiltrating NK cells play a predictive role in treatment efficacy (**Supplementary Figures S6**-**S7**). Although baseline PD-1 and PD-L1 expression on NK cells did not differ significantly between long-survival and short-survival groups, our previous research and current results demonstrate that an increased percentage of PD-L1+ NK cells after treatment is associated with significantly longer overall survival. Monitoring peripheral blood showed that patients with an increased post-treatment percentage of PD-L1+ NK cells had better outcomes compared to those with decreased percentages, indicating that the change in PD-L1+ NK cells could serve as a predictor of treatment efficacy. A previous study demonstrated that PD-L1 positive NK cells might be associated with enhanced NK cell function and improved patients’ survival (18). In contrast, the absence of a prognostic impact from changes in PD-1 expression on NK cells suggests that PD-1–mediated inhibition may play a less pivotal role in NK cell function in this context, or that other regulatory mechanisms are more influential post-treatment. Future research will explore the relationship between tumor-infiltrating NK cells and peripheral blood NK cells, as well as their impact on immunotherapy efficacy. Based on these efficacy-related biomarkers, we aim to select specific patient populations in future studies to clarify and validate their predictive value.

As we have previously mentioned in our article, the current study possesses certain limitations. Firstly, it is a single-center, single-arm clinical trial without a control group, which necessitates comparison solely with mono-immunotherapy data from similarly enrolled patients in the past. Secondly, owing to the exploratory nature of the study, the number of samples is limited. Lastly, although our study focused on its use in the second line setting, current data suggest that the regimen may have promising efficacy and a manageable safety profile. However, its feasibility as a first-line therapy requires further investigation in prospective, randomized clinical trials to directly compare outcomes with established first-line options. Additionally, there are also some challenges that remain. For future clinical studies, technical aspects will require optimization, including the dosage of NK cells infusions, the number of treatment cycles, and the identification of the benefit population. Furthermore, this study negates the predictive role of TMB in NK cells combined with PD-1 mAb therapy, while affirming the predictive role of tumor-infiltrating NK cells. The specific mechanisms of their actions still need to be clarified through further basic research.

In conclusion, the benefits outweigh the drawbacks, as this study presents the longest PFS and OS data reported thus far in second-line treatments for NSCLC without driver mutation genes (19–21). This provides a promising foundation for the future initiation of combined ICIs with NK cells therapy.

## Data Availability

The original contributions presented in the study are included in the article/**Supplementary Material**. Further inquiries can be directed to the corresponding author/s.
