# Supplementary material for: Updated overall survival data and predictive biomarkers of autologous NK cells plus Sintilimab as second-line treatment for advanced non-small cell lung cancer
Source: Front Immunol. 2025 May 21;16:1595382. doi: 10.3389/fimmu.2025.1595382 (PMC12133817; doi:10.3389/fimmu.2025.1595382)
Supplement: Supplementary file 1 [file DataSheet1.pdf]

Table S1

Subgroup	N, %	Median OS, months (95% CI)	HR (95% CI)	P value
Age, years				
≥ 65	5, 25%	NA (NA to NA)	0.42 (0.09 to 1.88)	0.26
< 65	15, 75%	17.73 (1.11 to 34.36)		
Smoking status				
Smoker	10, 50%	32.23 (6.71 to 57.75)	1.79 (0.62 to 5.17)	0.29
Never	10, 50%	15.20 (0 to 38.97)		
Histology				
Adenocarcinoma	8, 40%	7.47 (0 to 15.23)	0.57 (0.20 to 1.68)	0.31
Squamous	12, 60%	29.83 (21.41 to 38.25)		
TMB				
< 9 mut/Mb	14, 70%	17.73 (0 to 49.21)	0.65 (0.21 to 1.96)	0.65
≥ 9 mut/Mb	6, 30%	27.27 (0 to 55.48)		

Table S1 OS by Baseline Characteristic Subgroups.

Abbreviation: CI, confidence interval; NA, not applicable.

Table S2

	All Patients (N = 20)			
Event, No. (%)	Any Grade	Grade 1	Grade 2	Grade 3
Any event	20 (100)	8 (40)	9 (45)	3 (15)
Any event leading to discontinuation	2 (10)	0 (0)	2 (10)	0 (0)
Most frequent events (≥10%)				
Hypoalbuminemia	11 (55)	9 (45)	2 (10)	0 (0)
Anemia	10 (50)	8 (40)	2 (10)	0 (0)
Hypothyroidism	8 (40)	4 (20)	4 (20)	0 (0)
Hyperglycemia	7 (35)	5 (25)	1 (5)	1 (5)
Hypocalcemia	6 (30)	5 (25)	1 (5)	
Hyperuricemia	6 (30)	6 (30)		
LDL increased	6 (30)	6 (30)		
Luteinizing hormone increased	5 (25)	5 (25)		
Increased growth hormone	5 (25)	5 (25)		
Hyponatremia	5 (25)	5 (25)		
FSH increased	5 (25)	5 (25)		
Hypertriglyceridemia	4 (20)	3 (15)		1 (5)
Creatine kinase increased	4 (20)	1 (5)	2 (10)	1 (5)
Hyperthyroidism	4 (20)	3 (15)	1 (5)	
GGT increased	4 (20)	4 (20)		
Proteinuria	4 (20)	4 (20)		
Aspartate aminotransferase increased	4 (20)	4 (20)		
Prolactin increased	4 (20)	4 (20)		
Alanine aminotransferase increased	3 (15)	3 (15)		
Blood bilirubin increased	3 (15)	3 (15)		
Creatinine increased	3 (15)	3 (15)		
Alkaline phosphatase increased	3 (15)	3 (15)		
Lactic dehydrogenase increased	3 (15)	3 (15)		
White blood cell decreased	2 (10)	1 (5)		1 (5)
Neutrophil count decreased	2 (10)	1 (5)		1 (5)
Rash	2 (10)		2 (10)	
Luteinizing hormone decreased	2 (10)	2 (10)		
Creatine kinase isoenzyme increased	2 (10)	2 (10)		
Cough	2 (10)	2 (10)		
Platelet count decreased	2 (10)	2 (10)		

Table S2 Frequency of Adverse Events.

Table S3

**Subsequent Therapy**	**Disease progression patients (n=16)**
Any subsequent therapy	8 (50%)
Combined chemotherapy<br>Taxane and Platinum	4 (25%)
Tyrosine kinase inhibitor<br>Anlotinib	2 (12.5%)

Antibody-Drug Conjugate<br>Trastuzumab deruxtecan	1 (6.25%)
ICIs<br>CTLA-4 and PD-L1 antibodies	1 (6.25%)

Table S3 Subsequent Therapy.

Fig. S1

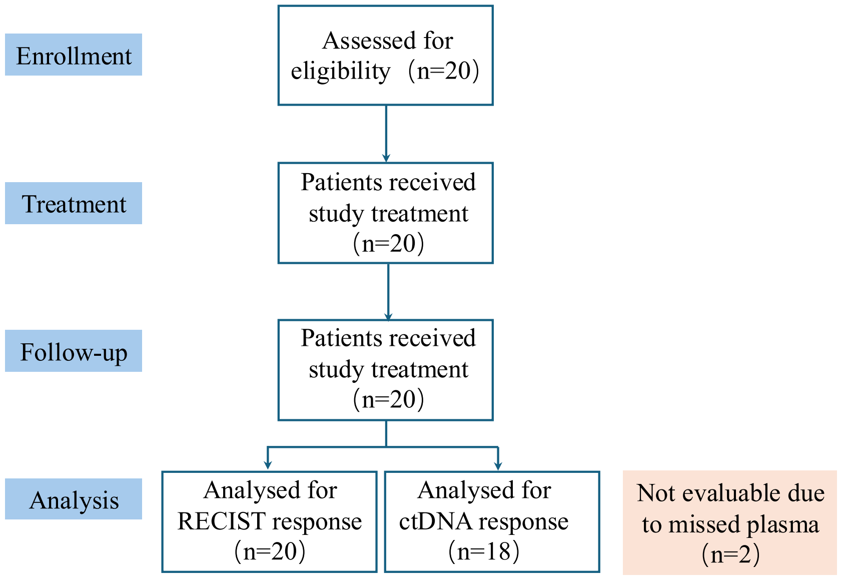

Fig. S1 CONSORT Flow Diagram for the Study.

Fig. S2

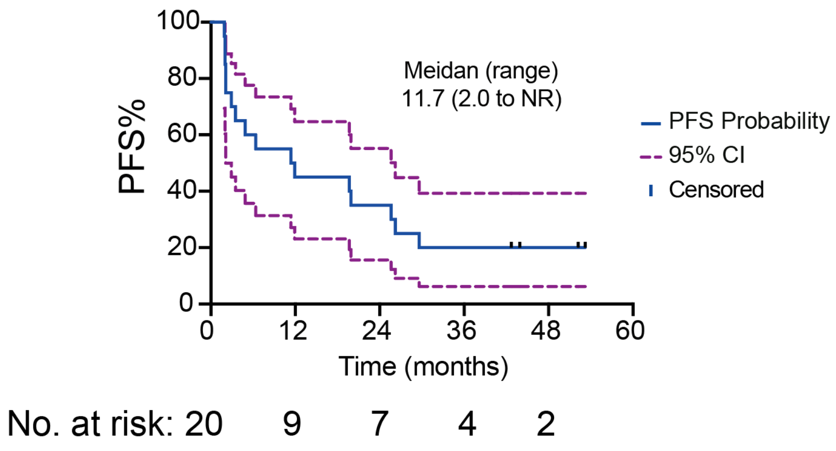

Fig. S2 Kaplan-Meier curve illustrating the median PFS and 95% CI for 20 patients. The solid blue line depicts the survival probability, while the dashed purple lines indicate the 95% CI. Tick marks represent censoring times. The numbers at risk correspond to the number of patients present at each time point. Abbreviation: NR, not reached.

Fig. S3

A. **B.**

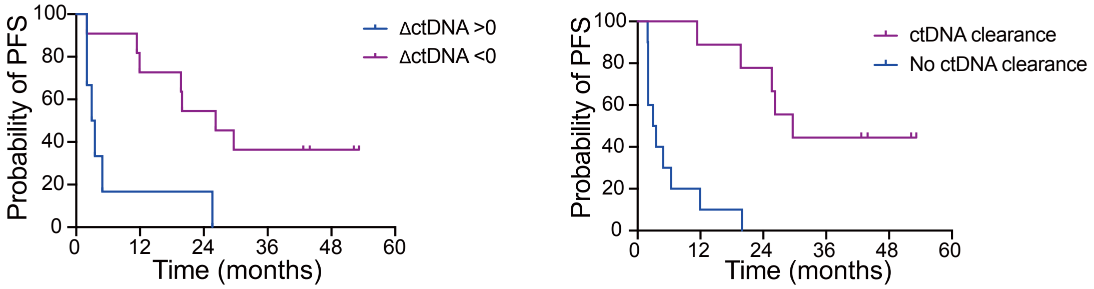

Fig. S3 Analyses of PFS by ctDNA dynamics. A. Patients exhibiting decreased ctDNA levels during therapy demonstrated longer PFS (26.2 vs. 3.2; HR=0.19 [95%CI: 0.06-0.67]; P=0.01). B. Patients achieved ctDNA clearance had longer PFS compared those who never achieved ctDNA clearance (29.6 vs. 3.2; HR=0.08 [95%CI: 0.02-0.41]; P =0.002).

Fig. S4

A.

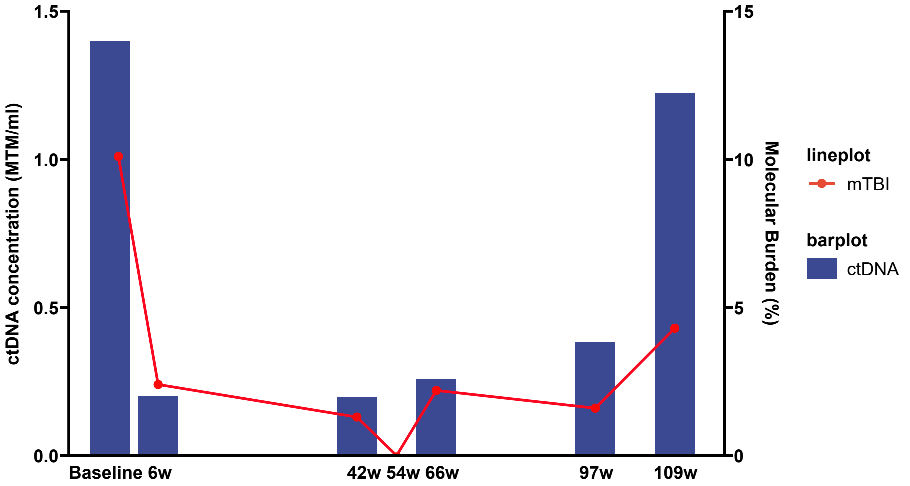

B.

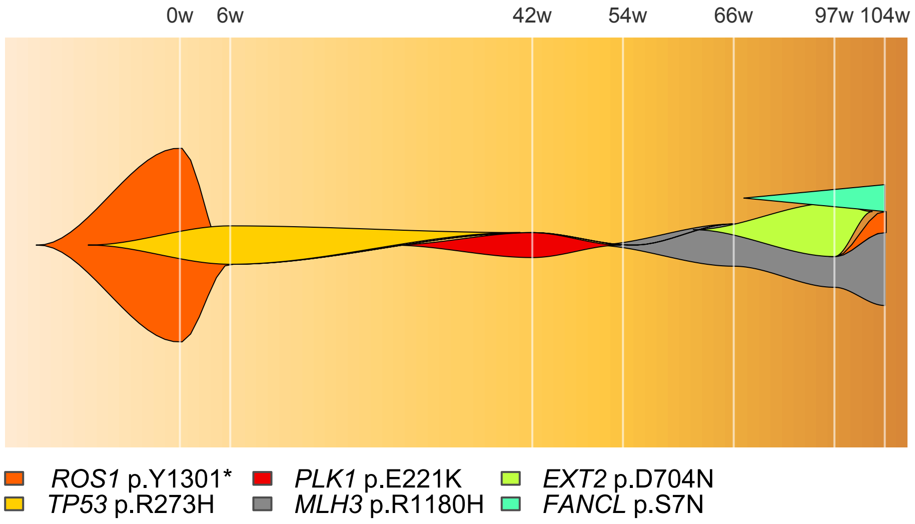

C.

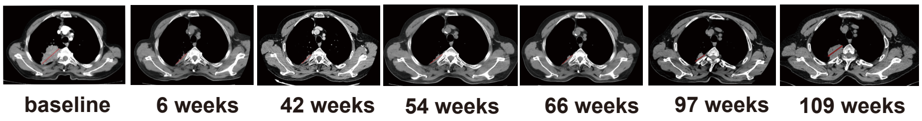

Fig. S4 Tracking tumor evolution and radiographic changes of patient 10009. At the 6-week tumor assessment, a significant reduction in tumor size was observed along with a notable decrease in ctDNA levels. The best response was observed at the 54-week tumor assessment, at which time ctDNA clearance was also achieved. However, at 97 weeks, an increase in ctDNA levels was detected, and by the 109-week tumor assessment, disease progression was confirmed. Clonal evolution analysis revealed emerging mutations in the EXT2, MLH3, and FANCL genes, and a re-emergence of a previously absent ROS1 gene mutation. **A.** Dynamic changes in ctDNA and mTBI during therapy. **B.** Diagram illustrating the evolution of gene cloning. **C.** Representative radiographic images at different time points.

Fig. S5

A. **B.**

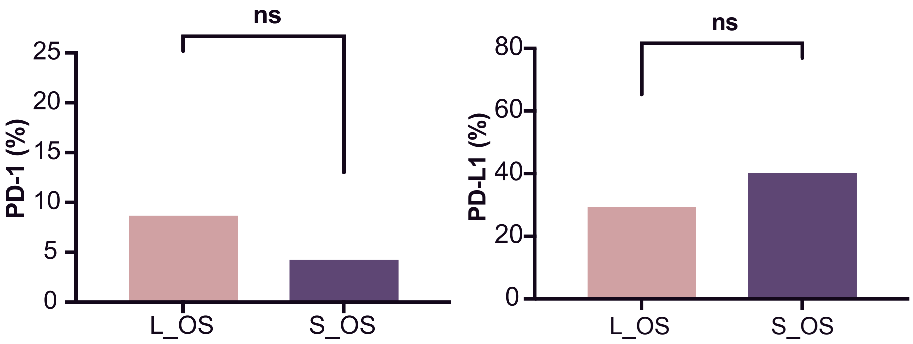

Fig. S5 Baseline PD-1 and PD-L1 expression on NK cells. A-B. There were no significant differences in baseline PD-1+ and PD-L1+ percentage of NK cells between the long-survival and short-survival groups. L_OS: long overall survival (>24months); S_OS: short overall survival (≤24 months)

Fig. S6

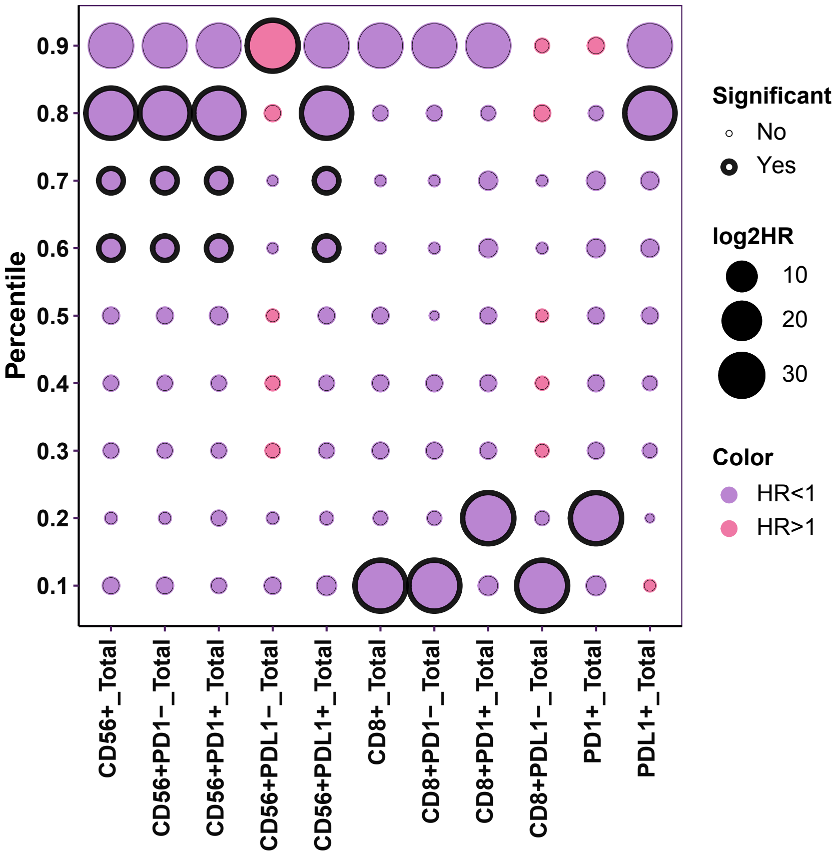

Fig. S6 Prognostic markers of OS with significance. Significant: P<0.05

Fig. S7

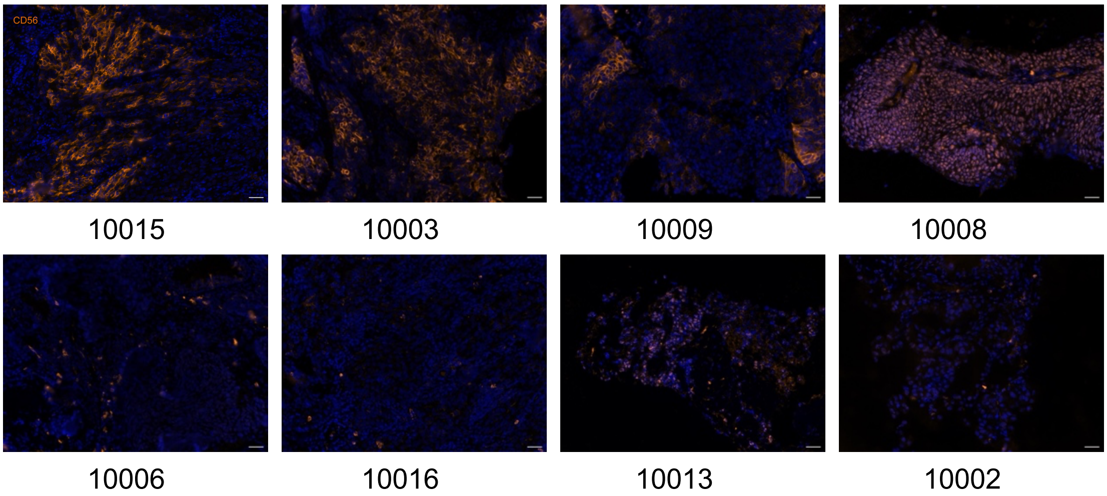

Fig. S7 Depiction of staining outcomes for CD56+ cells. BoR of 10015 was CR. BoR of 10003, 10009 was PR. BoR of 10008, 1006, 10016 was SD. BoR of 10002 was PD. A higher density of CD56+ cell infiltration correlated with more favorable therapeutic responses. Brown color indicates cells positive for CD56. Scale bar represents 100μm.

Fig. S8

A. **B.**

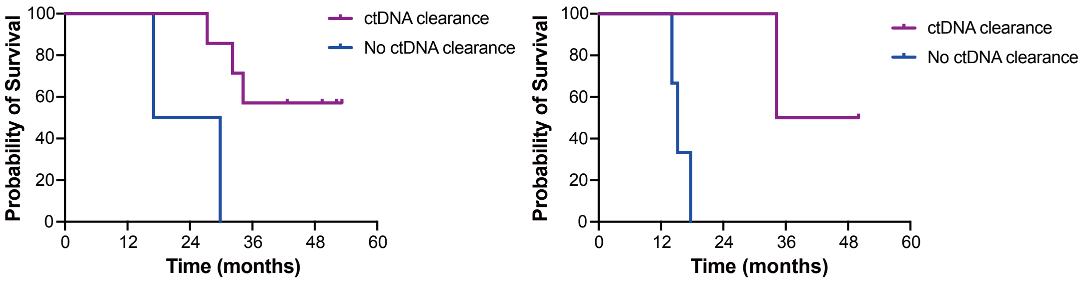

Fig. S8 Analyses of OS by ctDNA clearance in PR and SD patients. A. Patients achieved PR according to the RECIST v1.1 criteria, those who experienced ctDNA clearance had longer OS compared to those who did not achieve ctDNA clearance (NA vs. 23.4 months; HR=0.09 [95%CI: 0.01-1.05]; P=0.055). B. For patients with SD, the OS was longer in those who experienced ctDNA clearance (42.1 vs. 15.2 months; HR=0.01 [95%CI: 0.0-153.55]; P=0.360), although this difference was not statistically significant, likely due to the limited number of patients. NA, not applicable.
